# Circular Polarization of Transmitted Light by Sapphirinidae Copepods

**DOI:** 10.1371/journal.pone.0086131

**Published:** 2014-01-17

**Authors:** Yuval Baar, Joseph Rosen, Nadav Shashar

**Affiliations:** 1 Department of Life Sciences, Eilat Campus, Ben-Gurion University of the Negev, Beer-Sheva, Israel; 2 Department of Electrical and Computer Engineering, Ben-Gurion University of the Negev, Beer-Sheva, Israel; University of Sussex, United Kingdom

## Abstract

Circularly polarized light, rare in the animal kingdom, has thus far been documented in only a handful of animals. Using a rotating circular polarization (CP) analyzer we detected CP in linearly polarized light transmitted through epipelagic free living *Sapphirina metallina* copepods. Both left and right handedness of CP was detected, generated from specific organs of the animal's body, especially on the dorsal cephalosome and prosome. Such CP transmittance may be generated by phase retardance either in the muscle fibers or in the multilayer membrane structure found underneath the cuticle. Although the role, if any, played by circularly polarized light in Sapphirinidae has yet to be clarified, in other animals it was suggested to take part in mate choice, species recognition, and other forms of communication.

**Highlights:**

Planktonic Sapphirinidae copepods were found to circularly polarize the light passing through them. Circular polarization may be created by unique, multilayered features of the membrane structure found under their cuticle or by organized muscle fibers.

## Introduction

Ambient light underwater is partially polarized linearly to varying degrees at different depths [1], [2], but circular polarization (CP) has been observed occurring naturally only near the water surface [3], [4]. Among animals, the production of circularly polarized light is rare, yet was documented in the bioluminescence of *Photuris lucicrescens* and *P. versicolor* firefly larvae, in reflections from beetles of the Scarabaeidae family, in *Panulirus argus* lobsters, and in stomatopods (mantis shrimp) [5]–[14].

Circularly polarized light reflected by helical microfibril layers in the exocuticle of beetles belonging to the Scarabaeidae family is usually left handed [5]–[7], [10], [12], [13]. The multilayer structure that is responsible for the circularly polarized light in the scarab beetle *Plusiotis resplendens* can be treated as a three-dimensional diffraction grating. In that sense, while its effect on the polarization state of the light differs, the exocuticle diffracts light much as a multilayer dielectric grating does. Indeed, three-dimensional gratings can reflect light that is circularly or elliptically polarized [15]. In mantis shrimps, CP reflection has been observed in the keel of the male *Odontodactylus cultrifer* and is generated by two optical components. The first, a linear polarizer, is based on the ordered arrangement of dichroic carotenoid Astaxanthin molecules. The second component is a quarter-wave retarder, laid at a 45 degree angle to the linear component, which is assumed to comprise oriented calcite crystals [9], [16]. Mantis shrimps are also unique in that not only do they present a CP reflection, they can also see CP light [8].

Copepods from the family Sapphirinidae are widely distributed in the tropical and subtropical seas around the world, where they occupy the epipelagic zone of the waters. Yet Sapphirinidae is not one of the dominant families in the epipelagic zone. In the Gulf of Aqaba Sapphirina copepods, including *Sapphirina metallina* (Dana, 1849), are common, although their distribution appears to be patchy and varies by season. Due to the extraordinary co-occurrence of partial transparency and iridescent coloration in this species, scientists have shown interest in them since the nineteenth century. Unlike many other species of copepods that migrate from deep to shallow waters, Sapphirinidae copepods do not migrate vertically, but some species perform reverse migration, remaining near the surface during the day [17]–[19].

Among the most striking features of the *S. metallina* male is its iridescence, which is caused by a multilayered-membrane structure in epidermal cells of the dorsal integument [17]–[19]. Iridescence constitutes one characteristic of the species' sexual dimorphism, which also includes differences in body size, shape, color, and more. However Vassiere [20] did not find sexual dimorphism in Sapphirinidae eye structure although follow-up studies are still required.

## Results

**Figure 1:**
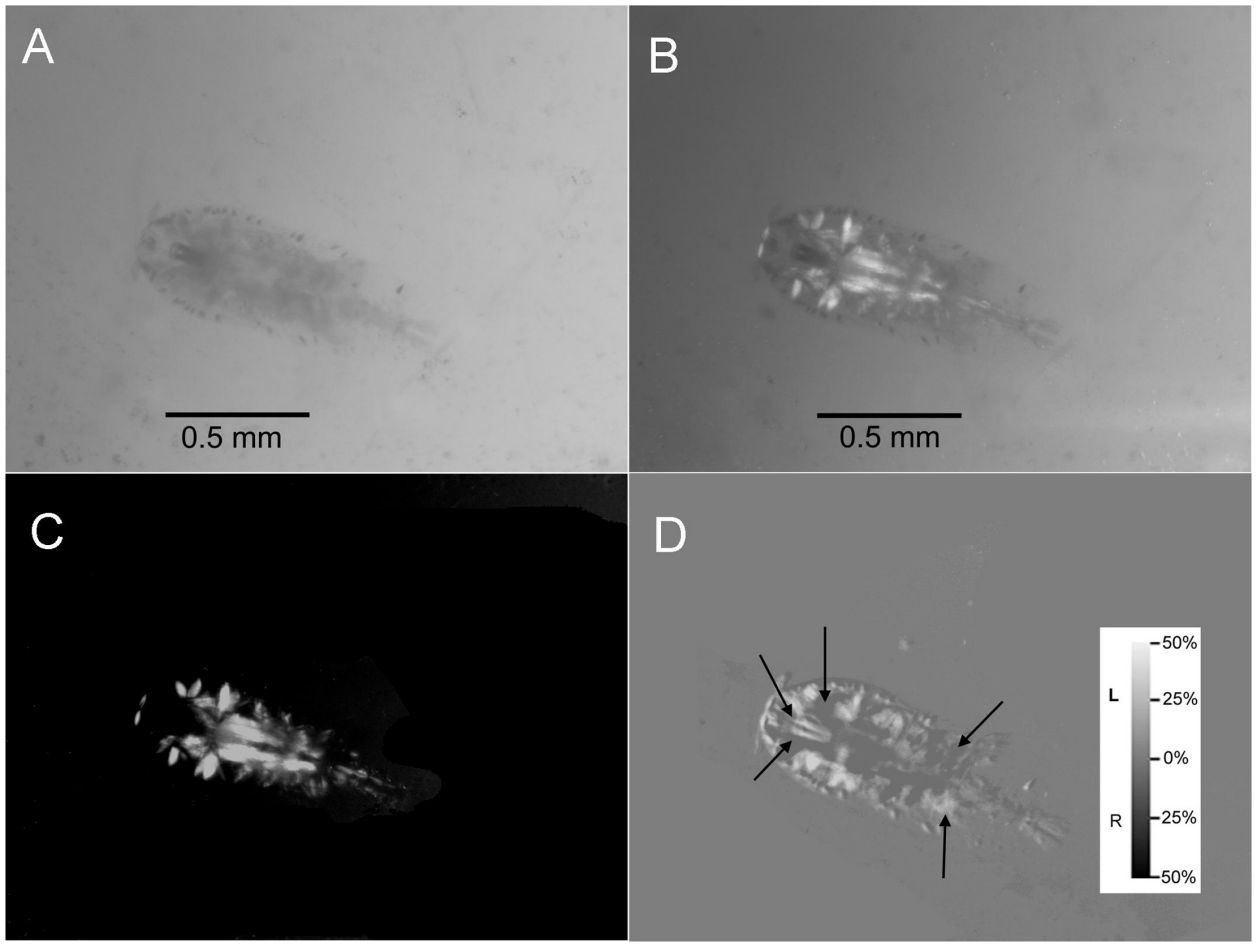
A *Sapphirina metallina* copepod under a dissecting microscope and transmitted illumination. **A**: With a depolarizing light and no polarizing filter. **B**: Between two linearly polarizers at 45° to each other, showing body structure and polarization active (depolarizing, phase retardance, or birefringence[24]) structures. **C**: The animal between crossed linear polarizers showing only linearly polarization-active structures. Such linear polarization activity can arise from change in orientation of polarization, depolarization, or the creation of CP. **D**: Circularly polarized light passing through a copepod, according to its left or right handedness. Incoming light was depolarized. Arrows indicate areas of relatively strong CP, though not more than 30%; dark and bright areas indicate right and left CP, respectively. Note that most of these areas, such as eye tubes or posterior parts of carapace, do not show up when placed between crossed linear polarizers (insert C) suggesting that the process causing the CP under depolarized illumination, is not mere retardance such as by muscle fibers.

**Figure 2:**
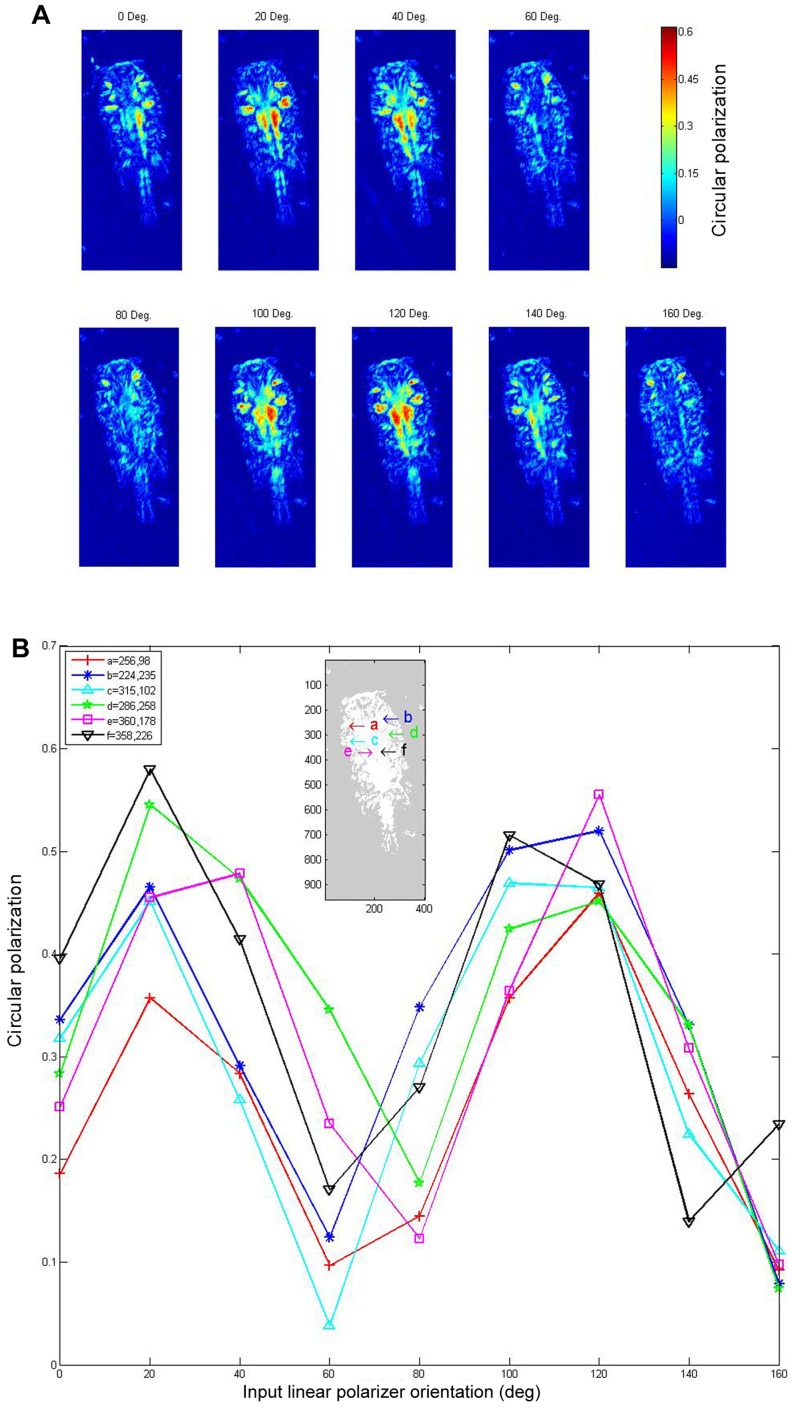
Maps of the modulation depth of the signals through a *Sapphirina* copepod, when incoming light is linearly polarized, as detected by a CP detector. A modulation depth of 1.0 indicates that all measured light is CP, whereas 0 signifies no CP. **A**: Each image is for a different orientation of the entrance polarizer, and therefore, of the illuminating beam, in 20° steps. The orientation for 0° was arbitrarily set. Note that CP handedness is not recorded. Locations of CP activity correspond with both muscles fibers as illuminated in Fig 1C and areas of CP created under depolarized illumination as in Fig 1D. **B**: The modulation depth as a function of the input polarization orientation at six locations on the animal is indicated in the figure inset.

*Sapphirina* copepods, which appear transparent to polarization-insensitive eyes, affected the linear polarization of light that passed through them, in the process partially circularly polarizing it. Due to the relative abundance of *S. metallina*, detailed analyses of CP focused on that species (Fig. 1). Figs. 1.B and 1.C demonstrate qualitatively that *S. metallina* has polarization activity with the linearly polarized transmitted light. The animal was rotated between two linear polarizers at 45° to each other (Fig. 1.B), and between crossed linear polarizers (Fig. 1.C), until an intensity change was detected. The brightness level at certain locations is about the same in both Figs 1.B and 1.C and this outcome is a clue that some level of CP exists herein. Fig. 1.D shows the result of the CP detector for depolarized illumination. The circular polarization effect was observed when the incoming light was either linearly polarized (Fig. 2) or depolarized (Fig. 1.D).

When the light striking the copepods was linearly polarized, the orientation of the polarization strongly affected CP. Fig. 2.A shows maps of the modulation depth of the signals as detected by the CP detector. Each image is for a different orientation of incoming light polarization in 20° steps. A modulation depth of 1.0 indicates that the all measured light was CP, whereas a depth of 0 signifies no CP. The modulation depth as a function of the input polarization angle at six locations on the animal, as indicated on the inset, is depicted in Fig. 2.B. CP levels, which were 30–60%, were maximal from the cephalosome, from the metasome, and in both lateral sides of the copepod's soma. The prominent regions are not identical in Figs. 1.D and 2.A because the effect of CP of the depolarized light is much weaker (Maximum CP value of 30%) than the CP created under linearly polarized illumination (Maximum CP value of 60%).

**Figure 3:**
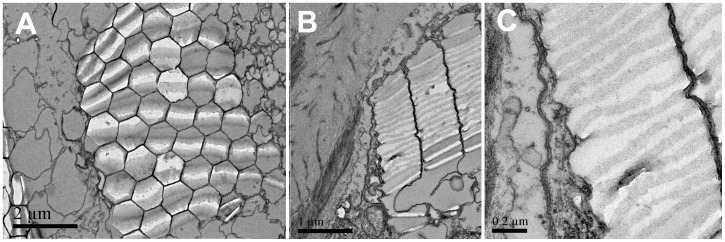
TEMs of *S. metallina*. **A**: frontal section of dorsal integument showing the multilayer membrane structure, a honeycomb arrangement, in which the first stack layer is parallel and some of the other layers are rotated on their sides showing a layered pattern. **B–C**: Sagittal sections of the membrane structure showing the two different membranes.

TEM images revealed the structure of the multilayer epidermal membrane (Fig. 3), showing structures that could serve as light retarders. Although we are not aware of any direct evidence of birefringence in these layered structures, we do not know of any mechanism other than retardation by birefringence to convert transmitted linear polarization to CP light.

In addition, in many cases the CP was transmitted from locations where muscle fibers affected the linear polarization (Fig. 1 B–D), suggesting retardance activity by these muscles.

## Discussion

Circularly polarized light in nature is currently known to be reflected from the cuticle tissues of marine crustaceans [9], [11] and of terrestrial arthropods [6], [10], [12], [13], [16].

In this work we present a case in which the light transmitted through an animals' body is circularly polarized. The effect was observed, to varying degrees, when the incoming illumination was either linearly polarized or depolarized.

The structural mechanism responsible for circular polarization in mantis shrimp and Scarabaeidae beetles is well understood [9], [13], [16]. In our case, since the strongest CP was obtained at specific input light polarization angles and in a cycle of 90°, it is clear that a part or parts of the Sapphirinidae copepod function as λ/4 retarder plates. At this point, however, we do not fully understand the underlying structural mechanism. Two mechanisms are possible: retardance by muscle fibers and/or by the multilayer membrane structure, the latter of which is also involved in Sapphirinidae body coloration. These mechanisms may be operating independently, or interacting and augmenting each other.

Muscles are known to be birefringent. The myosin-containing A bands of the sarcomere (the contractile unit) are birefringent [21], and indeed, light transmitted through vertebrate muscle tissues was demonstrated to undergo phase retardance [22], [23]. Sabhah and Shashar [24] demonstrated that muscle tissue changes the transmission of partially linearly polarized light passing through zooplankton. We propose here that the Sapphirinidae copepod circularly polarizes part of the linearly polarized light, thus contributing to its circularly polarized appearance.

Based on evidence from the optically active system in the copepods [17], which is responsible for their iridescent coloration, we suggest a model for how circular polarization is produced in these copepods. Their dorsal integument comprises a multilayered membrane made of 10–14 membrane pairs laid parallel to each other and to the cuticular plate. Viewed as a single structure, their dorsal integument has a regular hexagonal structure, and the gaps between the membrane layers contain a series of hexagonal platelets. Platelet thickness varies from 61–83 nm, and its estimated refractive index is 1.8 [17].

We hypothesize that the crystals in each layer of the multilayer hexagonal platelets are birefringent. If the optical path difference between the fast and the slow axes of the crystal is an odd number of quarter-wave lengths, then the multilayer hexagonal platelets are functioning as a quarter-wave retarder. Formally, the plate structure should follow the equation 2π·Δ*n*·*z*/*λ* = (2*N*+1)·π/2, where Δ*n* is the refractive index difference between the fast and the slow axes of the platelets, *z* is the overall thickness of the multilayer structure, *λ* is the average wavelength of the light, and *N* is an arbitrary positive whole number. Hence linearly polarized light (at a 45° angle to the fast and slow axes) transmitted through the copepods is circularly polarized. Note that the graph of the modulation depth of the CP detector signal as a function of the input polarization shows a cycle of π/2 as is expected from a quarter-wave retarder. Also note that most of cyclic signals changed together with almost no phase difference between them, an indication that all the crystals of the hexagonal platelets over the entire body of the copepod are oriented in approximately the same direction. However, we also found that the handedness of the circular polarization along the sides of the dorsal view (points a–d in Fig. 2B, inset) was opposite that in the central regions (points e–f in Fig. 2B, inset). This observation may indicate that the CP mechanism in each of these two regions is different. Further experimentation is needed to elucidate the retardance properties of the copepods.

In addition to the current partial understanding of the copepod phase retardance mechanism, the question remains of how non polarized light becomes circularly polarized, even if to a low extent, when passing through the animal's body. One possibility is that the incoming light is partially linearly polarized as it is refracted into or within the copepod (such as by eye tubes), especially when the light is arriving from directions that are not orthogonal to the curved body carapace. This partially linearly polarized light can then interact with the platelets to produce CP.

The role of circularly polarized light in the scarab beetle is not known [5]. Stomatopods use polarization in species-specific signals related to mating and defense [9]. It should be emphasized that some species of mantis shrimp, such as *Odontodactylus cultrifer*, can perceive and respond to CP signals, making them the only organisms with a known ability to sense circularly polarized light [8], [9].

Further research is needed to understand the role, if any, of the circularly polarized light transmitted through the body of Sapphirinidae copepods. The role of CP light transmission and their sensitivity to CP polarization should be examined both interspecifically, within the Sapphirinidae copepods including to examine for sexual dimorphism in the CP domain, and extraspecifically, for example, they may use CP light to avoid detection by predators sensitive to linear polarization. From the perspective of potential predators, it should be noted that stomatopod larvae are active planktonic predators.

## Materials and Methods

### Circular polarization measurements

**Figure 4:**
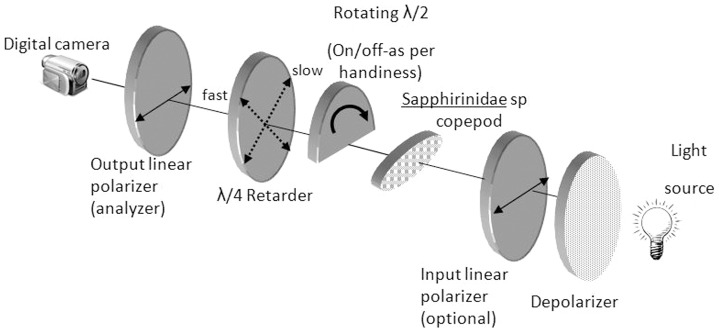
Outline of the system used to detect transmitted CP. Illuminating light passed first through a depolarizer, and then, if needed, it was linearly polarized. After passing through the specimen, depending on handedness, the light was or was not filtered through a rotating 1/2 λ retarder that covered half of the field of view. The circularly polarized light was then linearized by a 1/4 λ retarder and an analyzer was used to examine it.

For CP light detection, we built a system (Fig. 4) based on a Cokin® circular polarizing filter, half of which was covered from the direction of the incoming light by a λ/2 retarder (i.e., circular polarization handedness switching filter) with an optical path difference of 280 nm. A step motor rotated this apparatus sequentially to four positions at 90° intervals. At each position, the specimen was photographed via a Zeiss Stemi 2000-C polarization insensitive dissecting scope and a Nikon single CCD digital camera. To match the retarder filter (best fit for wavelengths around 560 nm), only the green channel of each image was used in further analysis. This approach resulted in four different pictures (orientations of the λ/2 retarder at 90° intervals) of the copepod.

### Polarization analysis

Circular polarization images were analyzed using a Matlab™ code to produce a numerical and visual representation of the differences in the light transmitted through the copepods as the analyzer was rotated. This was done by analyzing the modulation depth of the light signal detected along the rotation of the λ/2 plate. Note that our device is built to detect circularly polarized light, and to verify its reliability, it was tested under laser light (λ = 543 nm) in various known polarization states. In addition, the device was tested with a scarab beetle specimen, and a considerable, circularly polarized light signal was detected in the form of a periodic signal, as expected. The modulation depth was defined as (MAX−MIN)/(MAX+MIN), where MAX and MIN are the maximum and minimum intensities, respectively.

The pictures of the transmitted circularly polarized light include one for each of the four positions of the input λ/2 plate. Captured for the four different positions of the rotating λ/2 plate, the pictures represent four sampling points along a single cycle of the output signal. For every image pixel in each of the four pictures, the two values with the maximum intensity difference value (MAX−MIN) were chosen, and the difference between them was calculated. Each result was then divided by the sum of all the difference values (MAX+MIN) to give a value from 0–1. Defined as the modulation depth of the output cyclic signal, this value represents the percentage of the transmitted light that was circularly polarized from the total transmitted light.

### Animal collection

Copepods were collected by towing a 200 µm plankton net, just under the sea surface to depths up to 2 m, in the open waters of the Gulf of Aqaba, Eilat, Israel (following [25]). Over 20 *Sapphirina* copepods were collected for CP examination. More than half of them were *S. metallina* (Dana, 1849), and therefore, all further analysis focused exclusively on them.

### Electron microscopy

Specimen preparation for transmission electron microscopy followed [26] in detail. Freshly collected specimens were fixated in a 2.5% glutaraldehyde solution in a Cacodylate buffer at pH 7.4.

### EM preparation

Fixation and dehydration: Samples were fixed in the above for 3–24 h in the cold. Then they were rinsed 3 times for 10 minutes each in the same buffer, post-fixed in 1% Osmium tetroxide in the same buffer for 1 h and dehydrated in a graded ethanol series. During dehydration they were stained *en bloc* in uranyl acetate in 70% ethanol.

Embedding: Samples were embedded in Araldite resin. Embedding was gradual, using two 5-min rinses in epoxy propane (Propylene oxide) and then an hour each in increasing amounts of Araldite in epoxy propane (33%, 50%, 100%). Blocks were polymerized at 60° in an oven for 24 h.

Sectioning: Sections of various thicknesses (50–80 nm) were cut using a Leica Ultracut UCT ultra microtome (Leica Microsystems, Nussloch, Germany) and picked up on 75- or 100-mesh copper or nickel grids coated with Formvar and carbon. The sections were contrasted with uranyl acetate and lead citrate.

Transmission Electron Microscopy: Sections were observed in a Jeol JEM-1230 TEM (JEOL LTD, Tokyo, Japan) operated at 120 kV. Digital images were collected with a Gatan model 830 ORIUS SC200 CCD camera using Gatan's Digital Micrograph (DM) software.
